# Electrochemical Dye Switching Assisted Spectral Demixing and 3D STORM Imaging

**DOI:** 10.1002/anie.202517001

**Published:** 2026-03-20

**Authors:** Ying Yang, Yuanqing Ma, Justin Gooding

**Affiliations:** ^1^ School of Chemistry and Australian Centre For NanoMedicine University of New South Wales Sydney New South Wales Australia

**Keywords:** 3D localization microscopy, electrochemical dye switching, multicolor imaging, spectral demixing, super‐resolution microscopy

## Abstract

Super‐resolution microscopy has advanced cellular imaging by enabling nanoscale resolution, while reliable multicolor strategies remain crucial for resolving multiple targets in complex cellular samples. Here, we demonstrate a two‐color imaging strategy using electrochemically controlled stochastic optical reconstruction microscopy (EC‐STORM), which allows precise control over the switching behavior of two cyanine dyes, Alexa 647 and CF680. An oscillating electrochemical potential enables control of emitter density and molecule ON time. This minimizes the spatial and temporal overlap of emitters, enabling effective spectral demixing for two‐color EC‐STORM imaging. We further combined spectral demixing with a biplane detection configuration, enabling additional 3D localization of Alexa 647, where axial coordinates are extracted from the photon‐intensity ratio between the two detection channels instead of point spread function width. This integrated approach offers a practical solution for high‐density multicolor 3D STORM imaging.

## 1 Introduction

In conventional light microscopy, the spatial resolution is limited to approximately 200 nm due to light diffraction, restricting detailed visualization of subcellular structures. To overcome this limitation, a range of super‐resolution microscopy techniques have been developed, employing either optical engineering to manipulate the excitation and emission processes, or computational strategies to precisely localize single molecules [1, 2, 3, 4, 5, 6]. Among them, single‐molecule localization microscopy (SMLM) [4, 5, 6, 7], such as direct stochastic optical reconstruction microscopy (STORM), is one of the most widely used super‐resolution techniques. This is because SMLM achieves nanometer‐scale resolution with relatively simple instrumentation [6, 7]. The STORM technique overcomes the diffraction limit by temporally and spatially separating the emission of fluorophores, enabling the precise localization of individual molecules [6]. In each imaging frame, only a sparse subset of fluorophores is stochastically activated, allowing their positions to be determined with high precision by fitting their point spread functions (PSFs). Repeating this process across thousands of frames reconstructs a super‐resolved image from the accumulated localizations. While STORM has proven powerful for structural studies at the nanoscale, increasingly complex biological questions demand not only high resolution but also the ability to visualize multiple species simultaneously. This has driven growing interest in multicolor STORM, which enables spatial mapping of cellular organization and molecular interactions in complex systems. In parallel, capturing 3D positional information has become equally important, as many cellular structures and interactions are 3D. The development of 3D STORM techniques such as astigmatism‐based, biplane, and interferometric methods has expanded the capability of STORM to resolve structures in all three spatial dimensions, providing a more comprehensive view of subcellular architecture [8, 9, 10].

Early multicolor STORM approaches often relied on sequential acquisition, using spectrally distinct dyes, under different excitation/emission filter setups. While straightforward, imaging dyes with large spectral separations can introduce chromatic aberrations of up to ∼50 nm. This aberration can compromise any colocalization analysis [11]. Additionally, slower sequential acquisition increases sample drift, which can lead to additional registration errors [12]. To overcome the limitations of sequential acquisition, spectral demixing has emerged as a powerful alternative that enable simultaneous imaging. In this approach, fluorophores with partially overlapping emission spectra are excited by a single laser, and their emissions are split onto two cameras for synchronized detection. The relative intensity ratios between the two detection channels reflect the spectral signatures of individual dyes, enabling accurate assignment and separation. Spectral demixing minimizes chromatic aberrations and drift‐related errors, thereby supporting high‐quality multicolor super‐resolution imaging [13].

As an increasing number of organic dyes can now be photoswitched within a single imaging buffer, multicolor STORM based on spectral demixing has become widely used [14, 15, 16]. The resulting image quality, however, largely depends on the underlying photophysical properties of the fluorophores. One key factor is the duty cycle, which is the fraction of time a fluorophore remains in the fluorescent ON state and thus determines the effective emitter density during acquisition. Maintaining a low emitter density is essential to avoid overlap within diffraction‐limited areas. Common approaches to tune the duty cycle include adjusting laser intensities and buffer compositions [17, 18]. However, in densely labelled samples, the available range of adjustments becomes more constrained. For instance, laser intensity simultaneously influences both the off‐switching rate and reactivation efficiency, High thiol concentrations can reduce the emitter density but also lower photon output and hinders the recovery of fluorophores from dark states [18]. These interdependencies can make it challenging to identify imaging conditions that simultaneously suit multiple dyes. Strategies that provide finer control over the duty cycles and emitter density are therefore valuable for improving the consistency and performance of multicolor STORM imaging.

Our recent invention, electrochemically controlled STORM (EC‐STORM), offers a promising strategy for multicolor STORM imaging [19, 20, 21]. EC‐STORM introduces a new pathway for controlling the ON and OFF switching of STORM dyes by electrochemically tuning the underlying dye‐thiol reversible reaction. The applied potential modulates the redox environment, allowing the reversible switching process to be directed toward either the ON or OFF state. Using Alexa 647 as an example, it achieves nearly a 10‐fold increase in switching rate and expands the tunable duty cycle range by more than 50‐fold compared to conventional photochemical methods, thereby enabling robust control over emitter density for optimized STORM imaging. Building on these advances, we here explored the application of EC‐STORM to multicolor super‐resolution imaging. Specifically, we performed two‐color EC‐STORM using two far‐red cyanine dyes, AF647 and CF680, with a single excitation laser and standard STORM buffer in a dual‐camera demixing setup. The enhanced switching control afforded by electrochemistry enabled simultaneous super‐resolution imaging of microtubules and mitochondria. Furthermore, by incorporating a biplane detection scheme [9], we extracted both dual‐color and 3D localization information from the same dataset, demonstrating the versatility of EC‐STORM for advanced multicolor imaging applications.

## 2 Results and Discussion

### 2.1 Electrochemical Switching of Two STORM Dyes

**FIGURE 1:**
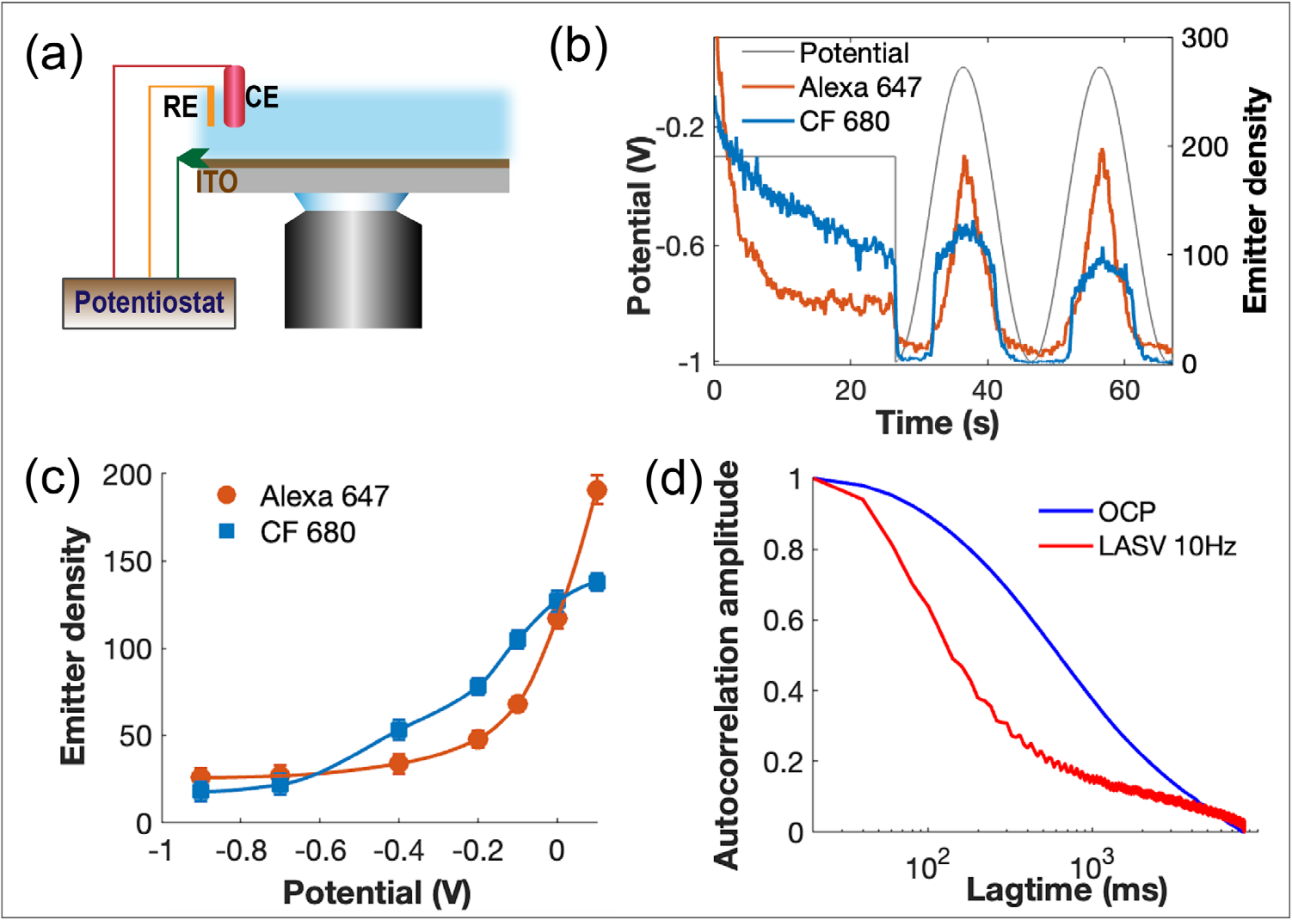
Electrochemical switching of Alexa 647 and CF680 dyes. (a) Schematic of the EC‐STORM imaging setup incorporating a three‐electrode system controlled via a potentiostat: ITO‐coated coverslip as both the working electrode and imaging substrate, Ag|AgCl|3 M KCl as the reference electrode (RE), and platinum mesh as the counter electrode (CE). (b) Plot of the dynamic changes in the detected emitter number of Alexa 647 and CF680 in response to large‐amplitude sinusoidal voltammetry, oscillating between −1 V and 0 V at 0.05 Hz. (c) Variation in the emitter density for Alexa 647 and CF 680 as a function of applied potential. (d) Autocorrelation analysis of CF680 fluorescence under OCP and LASV conditions. The OCP condition represents conventional STORM imaging using 642 nm laser excitation without any applied potential. In the LASV condition, the potential oscillated between −0.9 and −0.2 V at a frequency of 10 Hz. Alexa 647 tagged goat anti‐rabbit IgG and CF680 tagged goat anti‐mouse IgG were nonspecifically adsorbed onto separate ITO surfaces as imaging substrates, and a 1 kW cm^−^^2^ 642 nm laser was used for imaging.

As a first step, we tested whether CF680 can be reversibly switched ON and OFF using electrochemical potential as Alexa 647 can [20]. To enable electrochemical control during imaging, we used an indium tin oxide (ITO)‐coated glass coverslip as the working electrode [22, 23, 24, 25]. As shown in Figure 1a, a standard three‐electrode setup was integrated with a potentiostat for applying electrochemical potentials to the ITO surface. The conductive and optically transparent nature of ITO makes it well‐suited for high‐resolution imaging [24, 26, 27, 28]. We first imaged Alexa 647 and CF680 under 1 kW cm^−^^2^ 642 nm illumination at open‐circuit potential (OCP, −0.3 V), corresponding to conventional STORM conditions. Alexa 647 rapidly entered the OFF state, as expected under STORM imaging conditions, whereas CF680 exhibited slower and less efficient OFF switching, resulting in a much higher emitter density than Alexa 647. This mismatch in switching efficiency and emitter density illustrates a common challenge in two‐color STORM, where a single excitation and buffer condition is not equally optimal for both dyes.

To evaluate electrochemical control over dye behavior, we applied an oscillating potential between positive and negative potentials to the ITO surface. Both dyes responded reversibly, showing dynamic and reproducible switching. Alexa 647 maintained consistent switching across three cycles (Movie S1), whereas CF680 declined over time likely due to photobleaching. The tunability of emitter density by electrochemistry is further illustrated in Figure 1c, which plots emitter density versus potential. Alexa 647 showed a broad and reversible response, while CF680 was more sensitive to negative potential but less responsive to positive potential. These results demonstrate that electrochemical switching enables precise and reversible control over emitter density. This control will be particularly useful for two‐color STORM, where balancing emitter output is essential for uniform resolution and minimal crosstalk.

Building on the reversible control of emitter density shown in Figure 1c, we further show that electrochemical switching can also reduce the average ON time of dyes, which is critical for minimizing both spatial and temporal overlap of emitters within a diffraction‐limited area. This was achieved using large amplitude sinusoidal voltammetry (LASV), in which the applied potential oscillates between −0.9  and −0.2 V at a frequency of 10 Hz. The strongly negative potential efficiently switches dyes to the OFF state, while the less negative potential activates a small, stochastic subset of fluorophores to the ON state. The amplitude of the LASV, particularly the relatively positive potential, can be tuned to regulate the number of emitters per frame, enabling sparse activation conditions essential for resolving high‐density structures. We show this experimentally by comparing microtubule STORM images acquired with and without LASV in Figure S1, where reduced PSF overlap under LASV yields clearer reconstruction, most notably at filament crossing regions. More importantly, beyond controlling emitter density, this dynamic electrochemical oscillation significantly shortens the duration of ON events. As shown in Figure 1d, using CF680 as an example, the average ON‐time decreased from 600 ms under OCP (represents conventional STORM condition) to 140 ms with LASV, representing a 4‐fold reduction. The prolonged emission observed under OCP likely reflects extended ON‐states of CF680 molecules persisting across multiple frames. While a previous study using Alexa 647 demonstrated that LASV modulation effectively reduces ON time and regulates fluorescence intermittency [27], the present findings extend this strategy to CF680, further supporting the utility of EC switching for temporal control in multicolor EC‐STORM imaging.

### 2.2 Spectral Demixing of Alexa 647 and CF680

**FIGURE 2:**
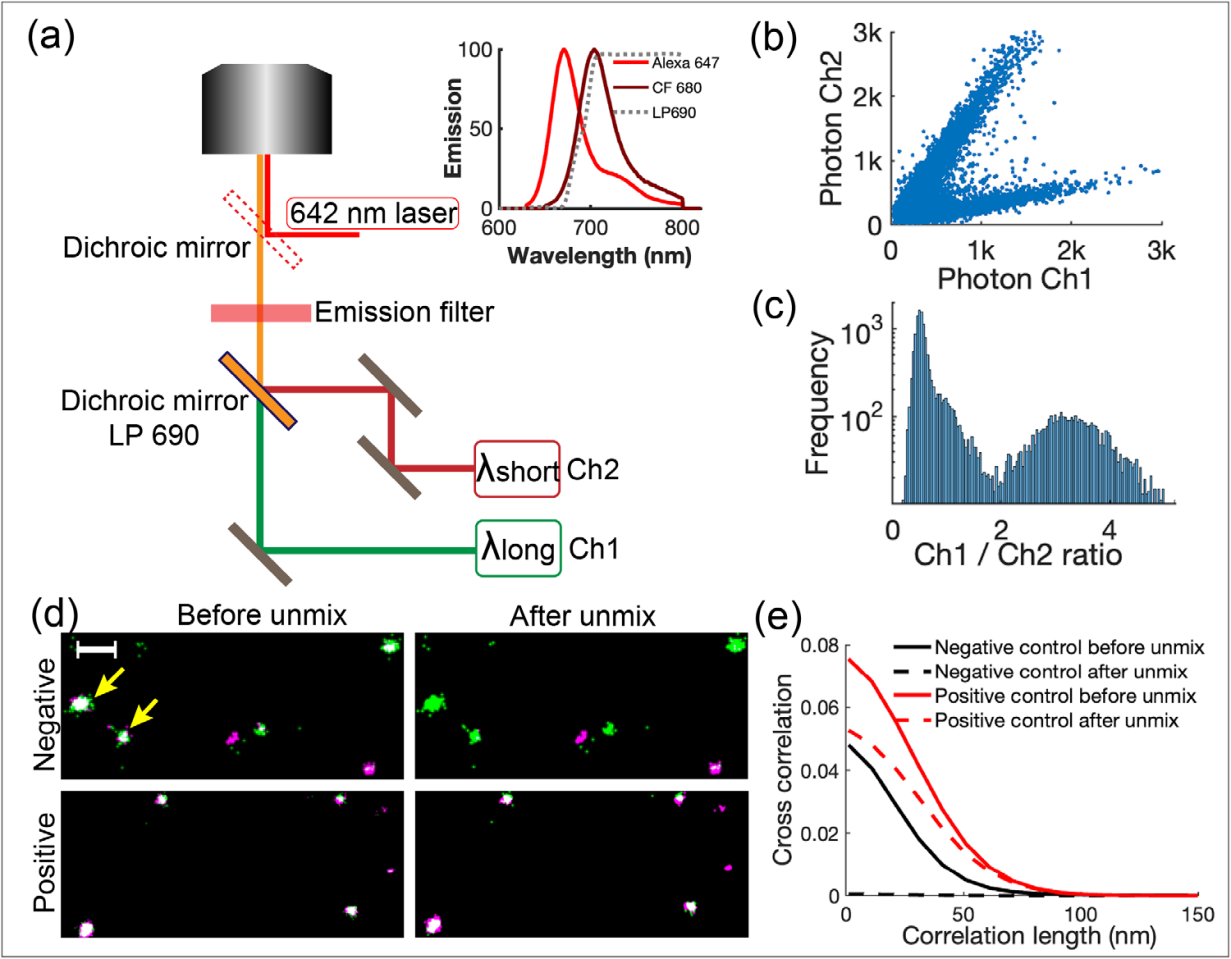
Spectral demixing of Alexa 647 and CF 680 for dual‐color EC‐STORM imaging. (a) Schematic of the dual‐camera TIRF microscope setup used for spectral demixing. A single 642 nm laser was used to excite both Alexa 647 and CF680 simultaneously. Emission was collected through a high‐NA oil‐immersion objective (100×, NA 1.49) and spectrally split by a 690 nm long‐pass dichroic mirror (LP690). The long‐wavelength emission (>690 nm) was directed to Camera 1 (Ch1), and the short‐wavelength emission (<690 nm) to Camera 2 (Ch2). Both cameras were synchronized for simultaneous image acquisition. The inset shows the normalized emission spectra of Alexa 647 and CF680 and the transmission profile of the LP690 filter. (b) Scatter plot of photon counts detected in Ch1 versus Ch2 for 17,951 single‐molecule localizations. (c) Histogram of the Ch1 to Ch2 photon ratio used for spectral classification of individual molecules. (d) Two‐color EC‐STORM images before (left) and after (right) spectral demixing. The top row shows a negative control (goat anti‐rabbit IgG–Alexa 647 and goat anti‐mouse IgG–CF680), and the bottom row shows a positive control (goat anti‐rabbit IgG–Alexa 647 and rabbit anti‐goat IgG–CF680). Antibody pairs were premixed in PBS for 1 h and immobilized on ITO‐coated coverslips via nonspecific adsorption. EC‐STORM imaging was performed over 5000 frames using 642 nm excitation under LASV condition (−0.9 to −0.2 V, 10 Hz); emission was split by a 690 nm long‐pass dichroic into Ch1 and Ch2. Single‐molecule localization was performed independently for each channel, followed by drift correction using cross‐correlation. Ch1 and Ch2 are pseudo‐colored magenta and green before demixing; Alexa 647 and CF680 are reassigned to green and magenta after demixing. Yellow arrows indicate Alexa 647 signals appearing in both channels prior to demixing. (e) Colocalization analysis of noninteracting and interacting antibody pairs via pair‐correlation. Scale bar = 200 nm in d.

Following optimize EC switching conditions for both Alexa 647 and CF680 under identical buffer conditions, we evaluated spectral demixing of the two dyes under the above LASV condition. As shown in Figure 2a, two‐color EC‐STORM imaging was performed using a TIRF microscope with a dual‐camera setup. Both Alexa 647 and CF680 were excited using a single 642 nm laser, and the emitted fluorescence was collected through the objective and subsequently spectrally split by a 690 nm long‐pass dichroic mirror. The long‐wavelength signal was transmitted to Channel 1 (Ch1), and the short‐wavelength signal was reflected to Channel 2 (Ch2), enabling simultaneous acquisition and spectral demixing. Although the emission spectra of Alexa 647 and CF680 overlap substantially, the minor spectral differences between the two dyes result in distinguishable signatures of photon distributions across the two channels, enabling effective spectral demixing [14, 16]. For Alexa 647, ∼60% of photons were detected in the short‐wavelength Ch2 and 40% in the long‐wavelength Ch1, while CF680 showed the opposite trend with 25% in Ch2 and 75% in Ch1. When the photon counts per molecule were plotted between the two channels, two distinct dye populations can be identified (Figure 2b). The linear scatter patterns of the two dye populations indicated that the fluorescence intensity ratio between channels is largely independent of molecular brightness, which can be affected by factors such as dye axial position or camera exposure time. A histogram of the Ch1 to Ch2 intensity ratio shows two well‐separated Gaussian distributions (Figure 2c), indicating that a single threshold allows for reliable distinction between the dyes under LASV condition.

To evaluate the reliability of ratiometric spectral demixing, we performed colocalization experiments using noninteracting and interacting antibody pairs labeled with Alexa 647 and CF680 (Figure 2d). Our results demonstrate that spectral crosstalk led to false colocalization between channels, which was effectively corrected by spectral demixing. In Figure 2d, molecules detected in Ch1 and Ch2 are pseudo‐colored magenta and green, respectively, with overlapping signals appearing white. In the negative control, where the antibodies are noninteracting, strong crosstalk from Alexa 647 resulted in numerous white spots prior to demixing, falsely suggesting colocalization (yellow arrows). Upon applying ratiometric thresholds at 2.0, channel crosstalk artifacts were effectively eliminated, resulting in clear separation of Alexa 647 and CF680 signals. In contrast, the positive control with the interacting antibody pair, a higher level of colocalization of the two dyes before demixing, as indicated by the presence of white clusters was observed. After demixing, these white signals remained largely unchanged, confirming that spectral demixing preserved genuine molecular interactions while eliminating crosstalk‐induced artifacts. We quantified the colocalization by the pair‐correlation approach [29, 30]. The cross‐correlation amplitude reflects the number of B molecules within a defined radius of each A molecule, while the correlation length represents the spatial scale of underlying molecular clusters, which in our case was ∼100 nm in diameter. In the negative control, spectral crosstalk resulted in a false cross‐correlation amplitude of 0.048, which was reduced to 0.001 after demixing. In the positive control, demixing corrected the false colocalization caused by spectral crosstalk, where the cross‐correlation amplitude decreased from 0.073 to 0.055. The remaining strong correlation reflects true antibody interaction. Thus, ratiometric thresholding of photon intensities across the two cameras enabled effective spectral demixing of Alexa 647 and CF680 under oscillating potentials.

### 2.3 Two‐Color 3D EC‐STORM Imaging

Once spectral demixing was validated on antibody‐coated surfaces, we next assessed its applicability for two‐color EC‐STORM imaging in cells. Achieving high spatial resolution in STORM requires careful regulation of emitter density to minimize the overlap of PSFs [19], a challenge that will be amplified when imaging with two spectrally overlapping dyes. Specifically, low emitter density per frame is essential to avoid spatial overlap of PSFs, while short fluorophore ON‐times would reduce temporal coincidence of fluorescent events within a diffraction‐limited region. As demonstrated in Figure 1d, LASV effectively shortens ON‐times and enables precise control of emitter density.

**FIGURE 3:**
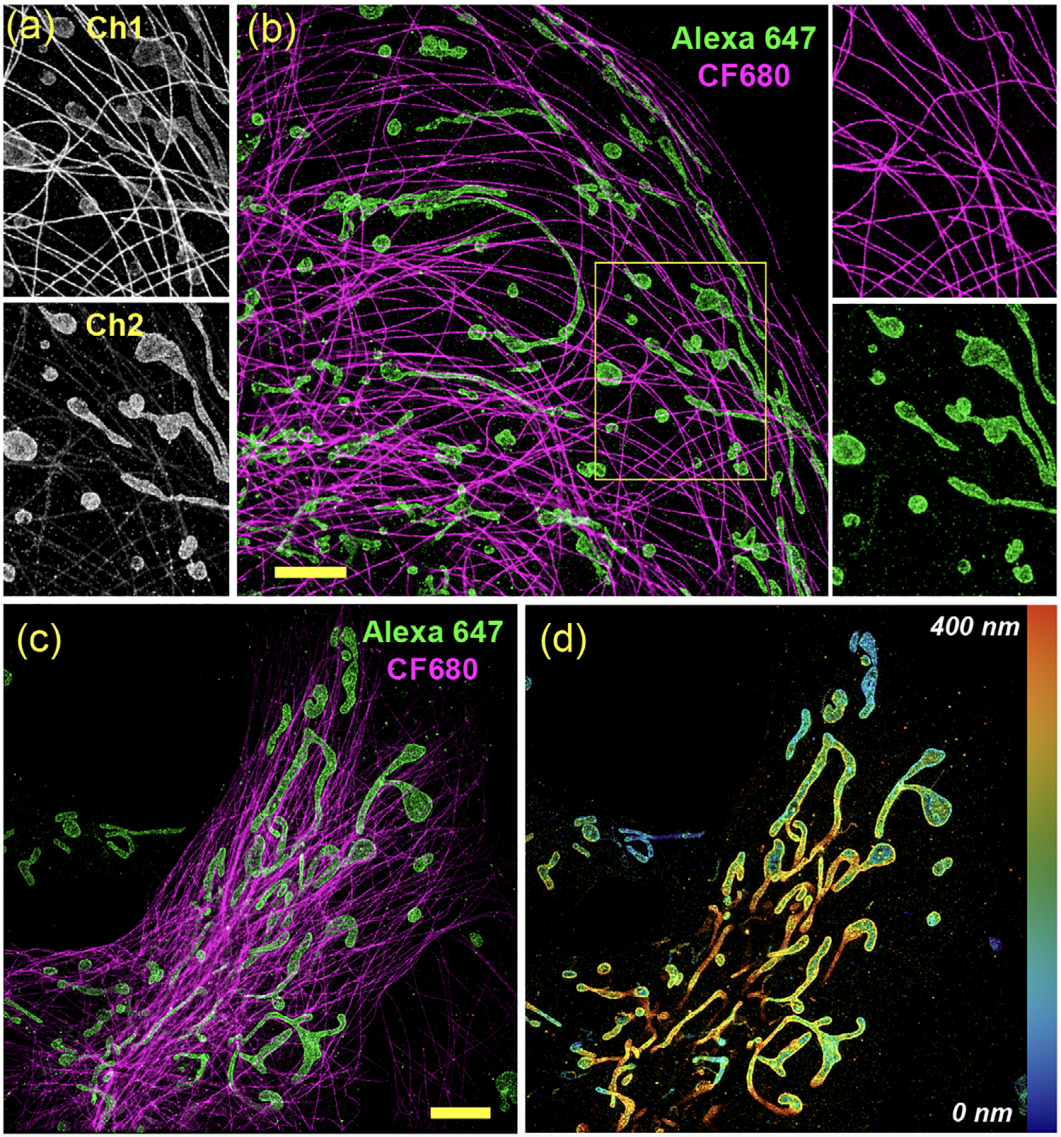
Two‐color and 3D EC‐STORM imaging. (a) Raw STORM images acquired in Ch1 and Ch2 prior to spectral demixing. Significant spectral crosstalk is observed, with pink signals from microtubules and the outer mitochondrial membrane TOM20 appearing in both channels due to emission overlap of Alexa 647 and CF680. (b) Two‐color EC‐STORM image of COS‐7 cells after spectral demixing, showing well‐separated microtubules labeled with Alexa 647 (green) TOM20 labeled with CF680 (magenta). The right panels show zoomed‐in, demixed views of the yellow‐boxed region, with each color channel displayed separately. (c) Spectrally demixed overlay of microtubules labeled with Alexa 647 and mitochondria labeled with CF680 acquired under the biplane detection setup. (d) Corresponding 3D EC‐STORM reconstruction of mitochondria from (c), with axial positions color‐coded from blue (0 nm) to red (400 nm), indicating increasing distance from the coverslip. Scale bars: 5 µm.

Building on these optimized electrochemical parameters, we applied two‐color EC‐STORM on microtubules and the outer mitochondrial membrane protein TOM20 in COS‐7 cells. Microtubules and TOM20 were labeled with CF680 and Alexa 647, respectively. Under fast LASV modulation, both dyes showed sparse emitter densities (Movie S2). Fluorescence time trace analysis suggests that the under LASV modulation, fluorescence events arise predominantly from single molecules, whereas high‐intensity spikes associated with multiple emitters within a diffraction‐limited spot are markedly suppressed (Figure S2). As shown in Figure 3a and Figure S3a, prior to spectral demixing, substantial crosstalk from Alexa 647 resulted in overlapping TOM20 signals appearing in both channels, CF680‐labeled microtubules were primarily detected in Ch1, with a small portion of signal also leaking into Ch2. After applying spectral demixing (Figure 3b), this crosstalk was effectively corrected, enabling a clear distinction between mitochondria and microtubules. We quantified the fluorescence intensity ratios across the two detection channels acquired in cell samples. The ratiometric analysis (Figure S3b) showed two distinct Gaussian populations, consistent with surface‐bound antibody samples, indicating that variations of molecular axial position within cellular structures do not affect the spectral demixing. Over 90% of CF680 molecules were detected exclusively in Ch1 and were therefore excluded from the ratiometric plot, but directly classified as CF680. Similarly, a small fraction of Alexa 647 molecules was detected only in Ch2 and directly identified as Alexa 647. Two‐channel localizations were demixed based on the fluorescence intensity ratio between Ch1 and Ch2, using threshold of 2.

Building upon our two color EC‐STORM setup, we further extended the system to 3D imaging by incorporating a biplane detection mode. This configuration not only enables dye separation through spectral demixing, but also allows axial (*z*) localization to be extracted from the same dataset [31]. In this setup, the focal plane of Ch2 shifted slightly above the focal plane of Ch1 by ∼ 600 nm, so the molecules near the coverslip appear defocused and dimmer in Ch2, whereas molecules located further from the coverslip are sharply focused and brighter in Ch2 but defocused and dimmer in Ch1. Therefore, either the PSF width or the photon intensity ratio between the two cameras can reflect the axial position of the molecules [31]. Extraction of PSF width during fitting requires low emitter density, as overlapping PSFs are typically excluded, which can lead to artifacts in dense areas of the reconstructed STORM image [32, 33]. Although LASV effectively decreased the overall emitter density and probability of PSF overlapping (Figure S4), PSF overlap can still occur in structurally dense regions. To overcome this, we routinely use a multi‐emitter fitting algorithm during PSF fitting in which the PSF width is fixed [32]. This causes the PSF width‐based axial localization unfeasible.

To address the limitation in multi‐emitter fitting, we evaluated whether the photon‐intensity ratio alone could be used for both dye demixing and axial determination. Under the biplane configuration, we performed STORM imaging of surface‐bound Alexa 647 and CF 680 molecules separately. To simulate the effect of axial position of the molecules to Ch1/Ch2 ratio, we moved the piezo stage at 10 nm step across 2.4 µm relative to the focal plane. As shown in Figure S5, Ch1/Ch2 ratio of Alexa 647 was mostly ≤ 2 regardless of its axial position. It exhibited a linear *z*‐dependent change in the Ch1/Ch2 ratio, decreasing from ∼2 to ∼ 0.8 at approximately ± 0.6 µm relative to the focal plane, which is below threshold for spectral demixing. There is a minor ∼ 2% of misclassification of Alexa 647 to CF 680 when it's located at 0.6 um below the focal plane. By contrast, CF 680 signals were detected predominantly in Ch1, so a reliable Ch1/Ch2 ratio could not be computed for most CF680 events. For the minority of CF680 localizations detected in both channels, the measured ratio was consistently ≥ 2 regardless its axial position. Accordingly, we used a coarse threshold of 2 for spectral demixing, and then used the finer *z*‐dependent Ch1/Ch2 ratio for axial localization of Alexa 647. As a result, 3D localization in the current configuration is feasible for Alexa 647, with an estimated axial resolution of ∼80 nm in the range of ± 0.6 µm relative to the focal plane (Figure S6), while CF680 is treated as a 2D channel.

We then performed two‐color 3D EC‐STORM image of microtubule and mitochondria, which were labelled by CF680 and Alexa 647, respectively. The spectral demixing and 3D localization were acquired from the same data set. From the spectral demixed two‐color image of Figure 3c, the respective cell components were clearly separated. The 3D image of mitochondria was reconstructed from the photon intensity ratio of the post‐spectral demixed Alexa 647 molecules. As shown in Figure 3d, there was no visible crosstalk from CF680‐labeled microtubules into the 3D image of Alexa 647‐labeled mitochondria, suggesting that our practical approach for dye classification and 3D localization using only the photon intensity ratio is feasible.

## 3 Conclusions

Controlling emitter density is critical for achieving accurate single‐molecule localization in STORM imaging. Conventional ways to optimize emitter density typically involve adjusting laser intensity, tuning buffer compositions such as oxygen and thiol concentrations, or carefully optimizing sample labeling strategies to control fluorophore density [17, 19, 34]. The development of electrochemical dye switching introduced a robust strategy to tune both emitter density and fluorescent ON‐time through applied potentials, thereby improving the performance of stochastic blinking‐based super‐resolution methods [20, 27]. For multicolor STORM with spectral demixing, spatial and temporal separation of emitters becomes increasingly critical, as overlap in space or time of different dyes can compromise the reliability of spectral demixing. To minimize temporal signal overlap, we introduced the use of an oscillating potential in this study, where the amplitude controls emitter density and the frequency dictates the average ON‐time of fluorophores [27], thereby increasing the fluorophore refreshing rate and reducing temporal overlap.

Using Alexa 647 and CF680 as model dyes, we demonstrated that oscillating potential enables dynamic ON–OFF switching of both fluorophores, supporting robust two‐color EC‐STORM imaging based on spectral demixing. In principle, the two‐channel ratiometric readout is not intrinsically limited to two dyes. With well‐characterized emission properties and high photon count, additional fluorophores may be separated after calibration by occupying distinct regions in the Ch1/Ch2 ratio space, as demonstrated in two‐camera multicolor single‐molecule imaging [35, 36] and discussed elsewhere [37]. By further integrating a biplane imaging configuration, we extended this approach to achieve two‐color 3D EC‐STORM imaging. Notably, we demonstrate the bifunctional role of photon intensity ratios for both spectral demixing and 3D localization. Previously, this can only be achieved by using both photon intensity ratio and PSF width, which requires single molecule fitting algorithm of low‐molecule‐density samples where overlapping emitters are discarded. Rejection of overlapping events occur in densely labeled regions and can lead to disrupt structural continuity [32, 33]. By extracting axial position directly from photon‐intensity ratios rather than PSF width, the present approach remains compatible with multi‐emitter fitting algorithms, enabling 3D localization in dense structures while preserving structural information. At present, however, EC‐STORM is primarily suited to fixed samples rather than live‐cell imaging, because the method requires electrochemical control and thiol‐containing STORM buffer conditions to support reversible dye switching. Looking ahead, the ability of EC‐STORM to combine precise electrochemical control with multi‐target imaging opens exciting opportunities to visualize molecular interactions and structural organization in complex cellular environments.

## Conflicts of Interest

The authors declare no conflicts of interest.

## Supporting information




**Supporting File: 1** anie71836‐sup‐0001‐SuppMat.docx.

**Supporting File: 2** anie71836‐sup‐0002‐MovieS1.mov.

**Supporting File: 3** anie71836‐sup‐0003‐MovieS2.mov.

## Data Availability

The data that support the findings of this study are available from the corresponding author upon reasonable request.
