# Supplementary material for: Electrochemical Dye Switching Assisted Spectral Demixing and 3D STORM Imaging
Source: Angew Chem Int Ed Engl. 2026 Mar 20;65(18):e17001. doi: 10.1002/anie.202517001 (PMC13110775; doi:10.1002/anie.202517001)
Supplement: Supplementary file 1 — Supporting File: 1 anie71836‐sup‐0001‐SuppMat.docx. [file ANIE-65-e17001-s002.docx]

**Supporting Information for**

**Electrochemical dye switching assisted spectral demixing and 3D STORM imaging**

Ying Yang^1^, Yuanqing Ma^1*^, Justin Gooding^1*^

^1^School of Chemistry and Australian Centre for NanoMedicine, University of New South Wales, Sydney, NSW 2052, Australia

Correspondence: yuanqing.ma@unsw.edu.au (Y.M.); justin.gooding@unsw.edu.au (J.G.)

**1. Method and materials**

1.1 Microscope setup

A Zeiss Elyra 7 Super-resolution microscope was used for the STORM data acquisition. The collimated and linearly p-polarized 642 nm laser was reflected from the 650 nm long pass dichroic mirror and focused on the edge of the back focal plane of the 100 X 1.46 NA Oil objective. The laser light became collimated and enters the ITO and water interface above the critical angle at 66.7^o^. For electrochemistry-based STORM, 1-4 kW cm^-2^ 642 nm laser is used. The fluorescence emitted from the evanescent field was collected by the same objective. After passing the 650 nm dichroic mirror, the fluorescence was split onto two sCMOS cameras (ORCA-Fusion Hamamatsu) through the Duolink system (Zeiss) by long pass dichroic beam splitter (690 nm, AHF Analysetechnik). A long focal length Tub lens in light path 2 of the DuoLink system allows for motorized XYZ movement for two channel alignment and biplane configuration.

For standard 2D imaging, the tube lens in path 2 was adjusted so that the images in both cameras are aligned and share the same focal plane. For biplane 3D imaging. The z position of tube lens in the path 2 was moved closer to the camera so that molecules located at ~600 nm higher in the sample space is focused on this camera. As a result, molecules from the lower focal plane are sharply focused on camera 1 but slightly out of focus on camera 2. Conversely, for molecules located in higher sample space would focus sharply on camera 2 and defocused on camera 1. Prior to two camera image acquisition, gold nanorods (A12-25-650-PAA-DUG-25, NanoParTz) was used as fiducial marker for two camera alignments. The nanoparticle has broad emission spectra that can be seen in both cameras.

During image acquisition, a hardware focus stabilization available for the Zeiss Elyra microscope was applied to minimize z drift. For 3D imaging, the objective was parked at the middle of the two focal planes to produce maximal contrast in the shape of PSF between the two cameras. A minimal of 50,000 images were acquired with exposure time of 20~35 ms with. The exposure time of the two cameras was synchronized by the internal trigger provided in the Zeiss Elyra. The localization fitting was done using the Zeiss Zen PALM software. Here, the signal to noise ratio of 6 and pixel radius of 9 was used to identify the molecules, and a 2D gaussian function was used to fit and localize the centroids of the molecules. Multi-emitter fitting algorithm was used for molecule fitting, where the PSF width was fixed to 160 nm. To compensate for the *xy* lateral drift occurred during image acquisition, the cross-correlation model-based based drift correction was applied.

1.2 Sample preparation

The ITO coated glass coverslip (06489-AB, SPI supplies, PA, USA) were commercially manufactured by depositing 750 nm ITO onto 170 μm thick glass coverslip that produce 8–12 Ω electric resistance according to the manufacture. The Ω value is indicative of the ITO thickness with the ITO coating thickness inversely proportional to electric resistance. Prior to placing samples on top, the ITO coverslip was plasma cleaned for 3 minute and washed by 70% ethanol.

For the two-colour antibody colocalization experiment, the goat anti rabbit IgG tagged with Alexa 647 (A-21235, Thermo Fisher), goat anti mouse IgG tagged with CF680 (20817, Biotium), and rabbit anti-goat IgG tagged with CF680 (20068, Biotium) was used. The antibody was premixed for 1 h at room temperature and added to the ITO surface to absorb by non-specific interactions.

COS-7 cells (ATCC CRL-1651) were cultured in Dulbecco′s Modified Eagle′s Medium containing 10% fetal bovine serum FBS, penicillin and streptomycin, and incubated at 37°C with 5% CO_2_. The cells were subculture onto ITO coverslip inside the 6-well plates at ~10,000-20,000 cells per well 12 h prior to fixation. The immunostaining procedure for microtubules consisted of: Fixation for 10 min at 37°C with 4% paraformaldehyde (Thermo Fisher), rinsing with PBS, permeabilization for 5 min with 0.2% Triton X-100. The cells were labelled by overnight incubation with respective primary antibody at ~ 2 µg mL^−1^ in blocking buffer (5% BSA in PBS). For two colour microtubule and mitochondria imaging, Rabbit anti α-tubulin monoclonal antibody (ab216650, Abcam) and mouse Tom20 antibody F-10 (sc-17764, Santa Cruz Biotechnology) was used. The cells were washed 3 times with PBS containing 0.2% Tween 20, then incubated for 30 min with corresponding secondary antibodies, which are goat anti-rabbit IgG tagged with CF680 (SAB4600362, Sigma) and goat anti-mouse IgG tagged with Alexa 647 (A-21235, Thermo Fisher).

1.3 Data analysis

The fitted STORM localization table contains the lateral coordinates (x, y), channel number, frame number, and the amplitude and width of the best-fit Gaussian function for each detected molecule. Localization fitting was performed in ZEN Blue (v3.11) using the “account for overlapping” option to preserve localization yield and structural continuity in dense regions. In this routine, only spots with a signal-to-noise ratio (SNR) > 6 were included in the fitting process, while dim spots below this threshold were excluded, and the PSF width is fixed to 160 nm.

Although the emission spectra of Alexa 647 and CF680 are closely spaced, Alexa 647 is blue-shifted relative to CF680. Accordingly, Alexa 647 contributes a larger fraction of photons to the short-wavelength channel (channel 2, 650–690 nm) and a smaller fraction to the long-wavelength channel (channel 1, 690–750 nm), whereas CF680 is predominantly detected in channel 1. Therefore, the photon ratio Ichannel 1/Ichannel 2 is typically < 2 for Alexa 647 and > 2 for CF680. Owing to the SNR threshold and unequal photon partitioning between the two channels, some molecules were detected only in their photon-dominant channel and did not produce a measurable corresponding intensity in the counterpart channel. For instance, most CF680 localizations were detected only in the longer-wavelength channel 1, and a smaller fraction of Alexa 647 localizations were detected only in channel 2. Based on this behaviour, these single-channel localizations were directly assigned to the corresponding fluorophore without ratio-based classification.

Accurate extraction of ratiometric values across the two channels requires identification of the same molecule in both channels within the same frame. This, in turn, requires high-precision alignment of the spatial coordinates between the channels. Although gold nanorods were used for two-channel alignment prior to image acquisition, sub-pixel level misalignment was difficult to correct visually. Therefore, the two-channel registration was further refined by cross-correlation in Fourier space using the reconstructed STORM images from the two channels. The relative lateral shift of the central cross-correlation peak in Fourier space was used to determine the translational x–y offset in real space, and this offset was applied for channel registration.

Once the two-channel alignment was complete, inter-channel pairing was performed on a frame-by-frame basis using a spatial-binning strategy. Candidate localizations from the two channels were first grouped within 250 nm square bins. If molecules were detected in both channels within the same 250 nm square bin in the same frame, they were regarded as photon emission from the same molecule across the two cameras. Bins containing exactly one localization in each channel were treated as unambiguous one-to-one pairs, and the photon ratiometric value between the two cameras was calculated from the fitted amplitudes and registered for spectral demixing and 3D localization. If more than one localization was present in either channel within the same bin, the bin was recursively subdivided into 62 nm bins and the pairing procedure was repeated.

1.4 Validation of ratiometric z localization in biplane imaging

To assess the impact of the fixed-PSF-width multi-emitter fitting used in the main analysis, we performed single-color AF647 z-scan calibration experiments under the same biplane imaging configuration as used in this study (focal-plane separation between Camera 1 and Camera 2: 600 nm). The piezo stage was stepped in 10 nm increments over a 2.4 µm axial range spanning the focal plane.

For this validation experiment, localizations were fitted using the ZEN Blue “discard overlapping” routine, in which the emitter x/y position, amplitude, and PSF width were fitted variables. We then compared z-dependent metrics based on PSF width [PSF width in Ch1 and PSF-width ratio (Ch1/Ch2)] with the photon-intensity ratio metric [Ch1/Ch2], which is used for axial localization in the main text (Supplementary Figure 6a–c).

Axial localization precision was estimated from the standard deviation of the Ch1/Ch2 photon-intensity ratio at each z plane and converted to the corresponding axial uncertainty using the calibration curve (Supplementary Figure 6d). The resulting precision is z-dependent, with <80 nm axial precision achieved on average within ±0.5 µm of focus.


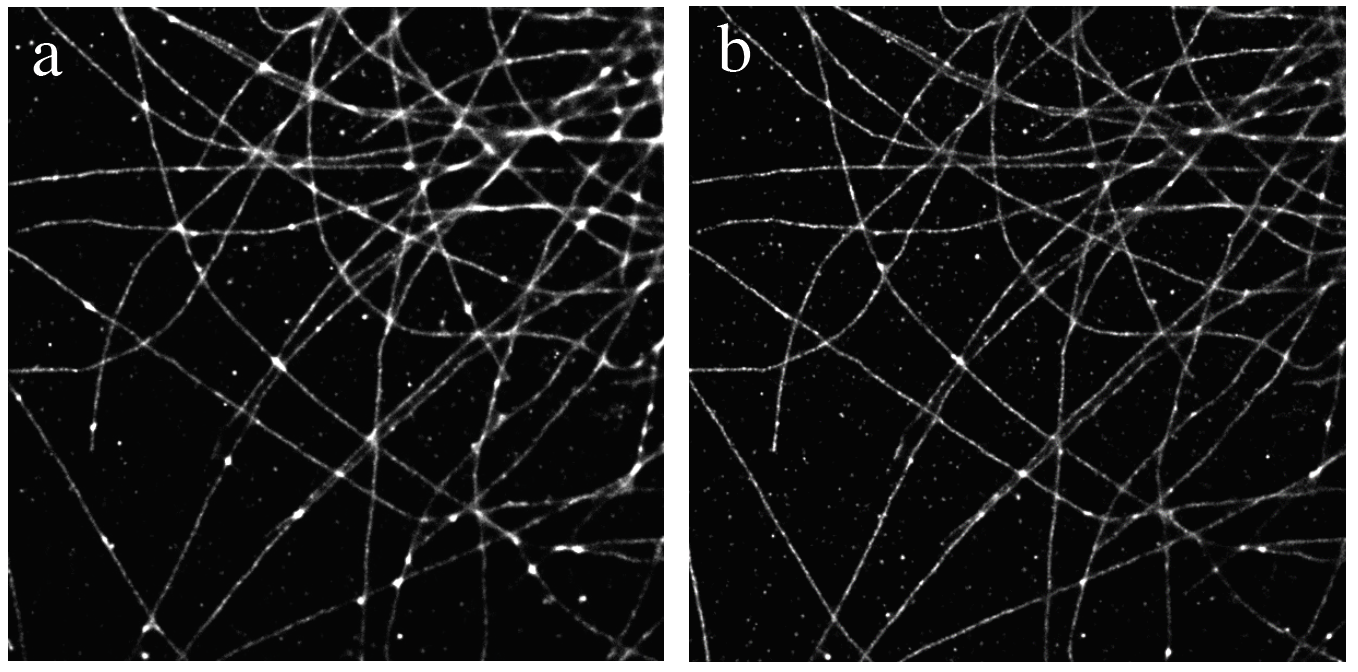


**Figure S1.** STORM and EC-STORM of the same Alexa 647 labelled tubulin sample. **(a)** Conventional STORM acquired with a 642-nm laser (2 kW cm⁻²) without UV activation. **(b)** EC-STORM acquired under same 642 nm laser illumination while applying LASV. The electrochemical potential was osilated between −0.6 and −0.4 V at 10 Hz. 10 K frames of images were recorded for each condition, while EC-STORM shows improved separation in crossing microtubules, with fewer blurred intersections.


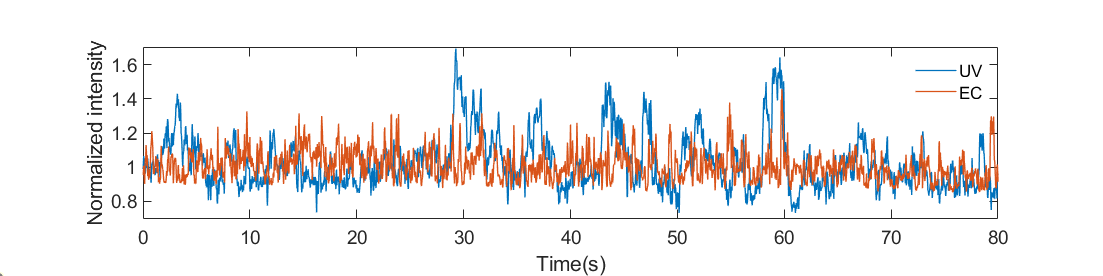


**Figure S2.** Fluorescence intensity time trace from the same pixel on an CF 680 labelled tubulin filament under conventional STORM and EC-STORM conditions. UV-STORM used a 642 nm excitation laser at 2 kW cm⁻². EC-STORM used the same excitation and LASV electrochemical modulation at 10 Hz between −0.6 and −0.4 V. Large and broad intensity bursts under UV-STORM suggest coincident emission from multiple emitters within a diffraction-limited spot, which is reduced under EC-STORM modulation.


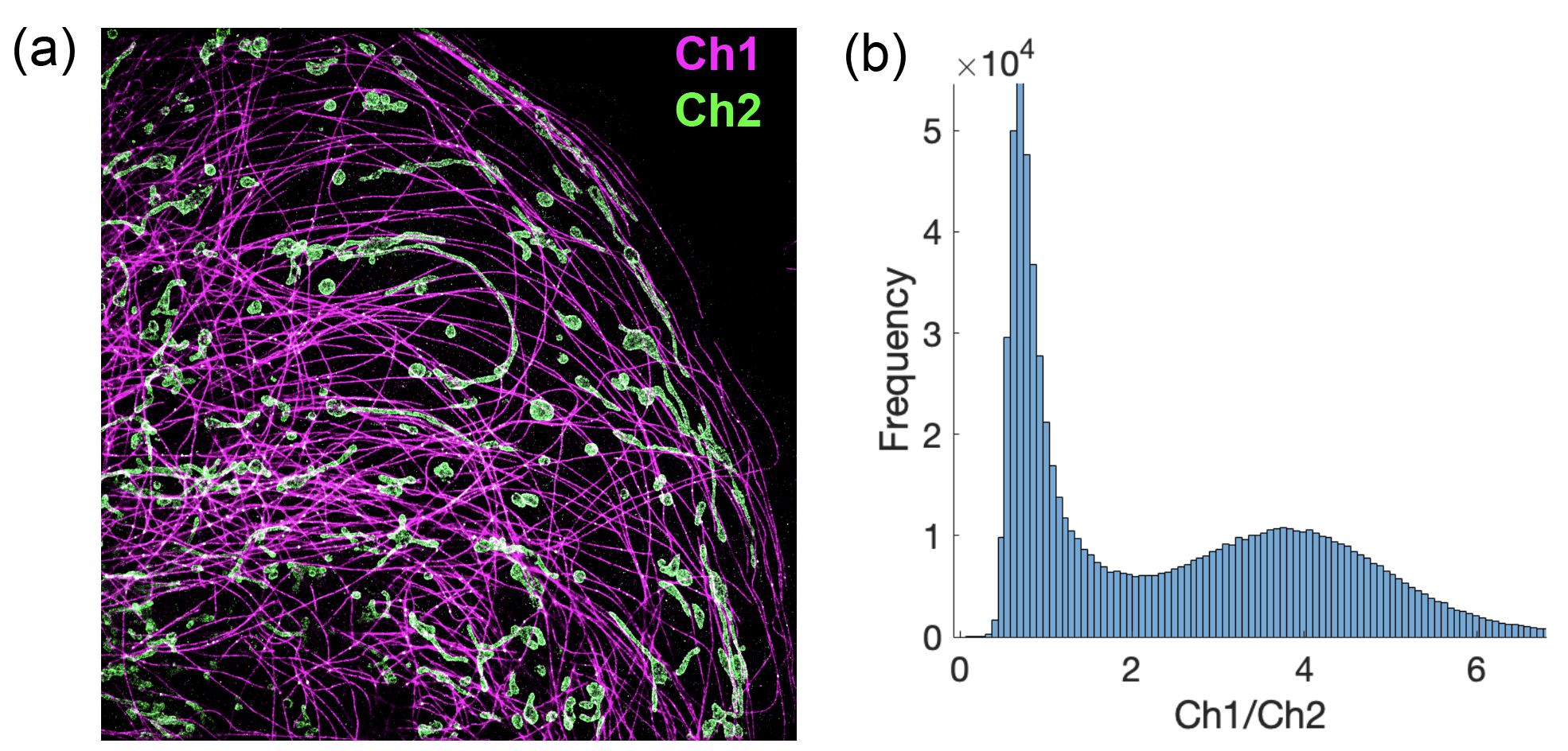


**Figure S3.** **(a)** Prior to spectral demixing, both Alexa 647 and CF680 signals were detected across the two channels. When Ch1 (magenta) and Ch2 (green) were merged, this spectral overlap caused structures to appear in both channels. Notably, substantial crosstalk from Alexa 647-labeled TOM20 resulted in a partially white appearance of the outer mitochondrial membrane. **(b)** Histogram of fluorescence intensity ratios (Ch1/Ch2) measured from cellular samples, revealing two distinct populations.


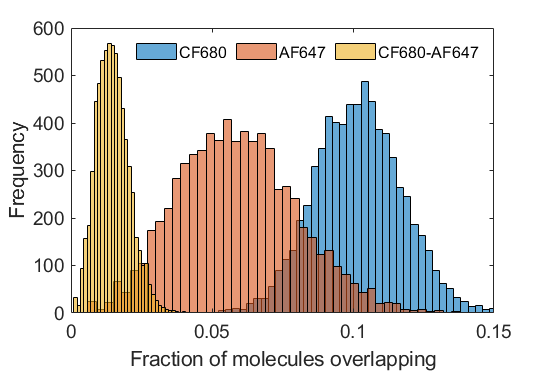


**Figure S4.** Fraction of overlapping localizations in the demixed EC-STORM dataset. Overlap was defined as an inter-molecular distance <300 nm. Euclidean distances were computed between all pairs of localizations in the demixed molecule list for CF680–CF680, AF647–AF647, and CF680–AF647 using the same cell region as Fig. 3a.


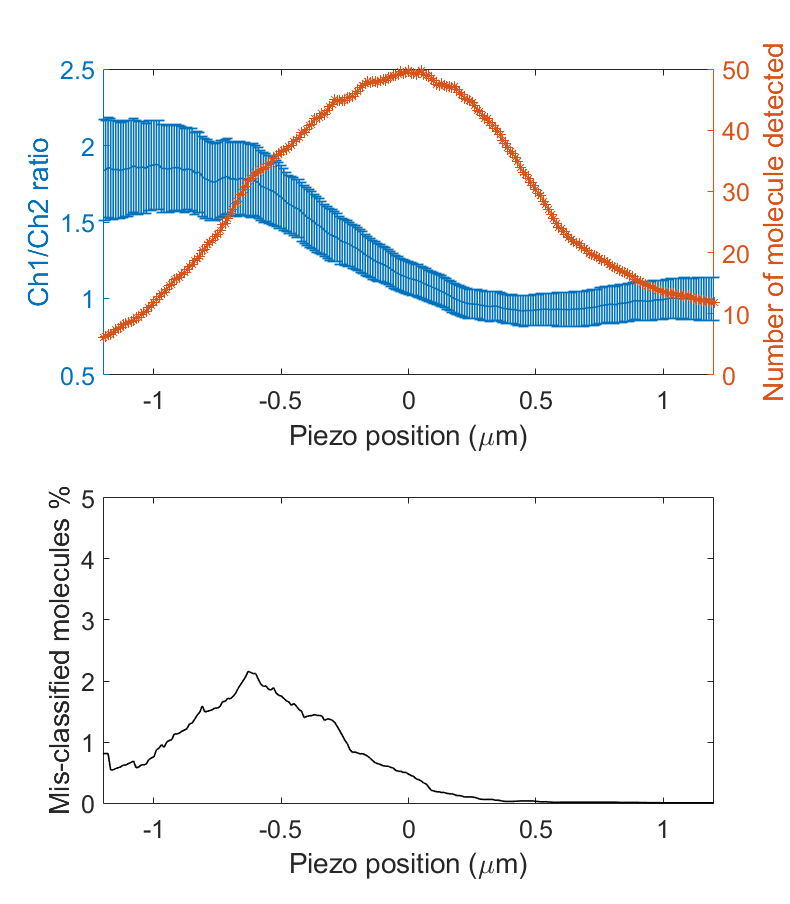

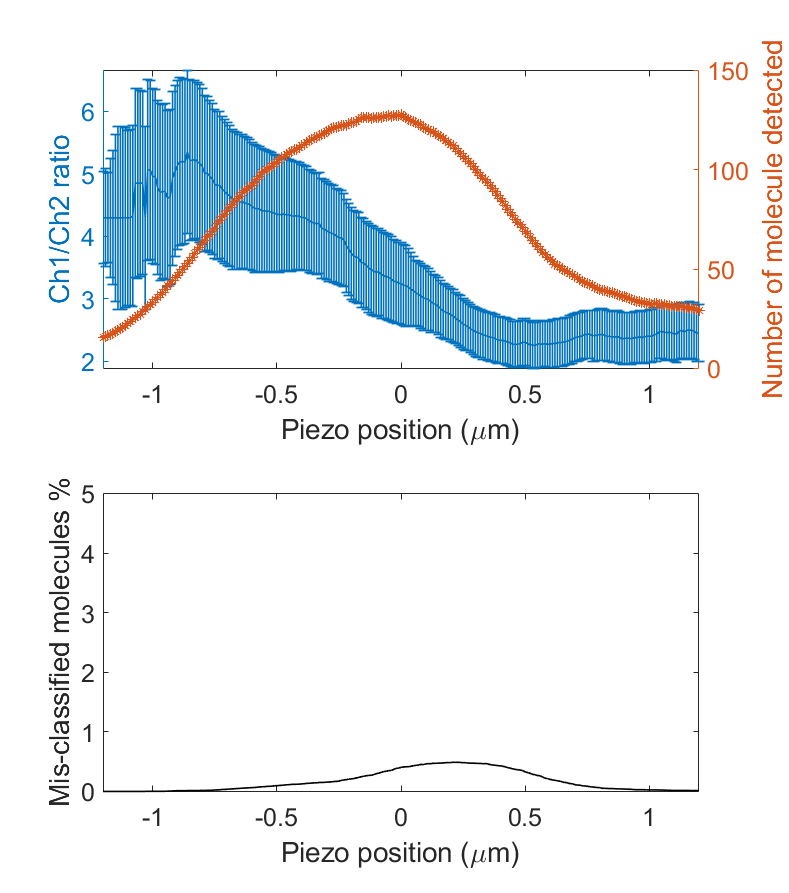


**Figure S5.** Ch1/Ch2 photon-intensity ratios of Alexa 647 and CF 680 under biplane imaging. Ch1/Ch2 photon-intensity ratios measured for Alexa 647 (left) and CF 680 (right) under biplane settings, where the focal planes of Camera 1 and Camera 2 were separated by 600 nm. Alexa 647 or CF 680 labelled antibodies were physically-adsorbed onto an ITO-coated coverslip and imaged in STORM buffer. A z-scan was performed by moving the piezo stage in 10 nm steps over a 2.4 µm range crossing the central focal plane. Localizations were obtained using a multi-emitter fitting algorithm, in which the x/y positions and PSF amplitudes were fitted while the PSF width was fixed at 160 nm. Misclassified events were defined as Alexa 647 localizations with Ch1/Ch2 > 2 and CF 680 localizations with Ch1/Ch2 < 2. The misclassification rate was weighted by the z-dependent detection efficiency, which was approximated by a Gaussian profile centered at the focal plane, reflecting the reduced detection probability as emitters moved out of focus.


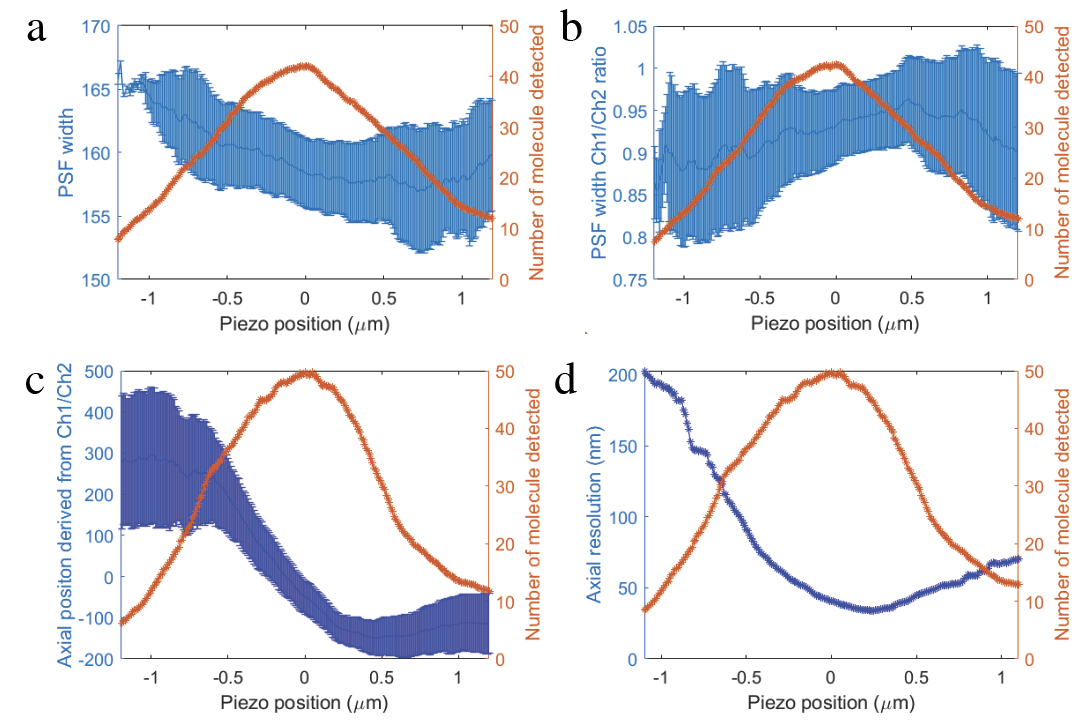


**Figure S6.** Single-color AF647 z-scan calibrations were performed under the same biplane settings used in this study, with the focal planes of Camera 1 and Camera 2 separated by 600 nm. The piezo stage was stepped in 10 nm increments over a 2.4 µm range spanning the focal plane. Localizations were fitted using the “discard overlapping” algorithm (Gaussian PSF model), in which the emitter x/y position, PSF amplitude, and PSF width were fitted. Panels show **(a)** PSF width in Ch1 versus piezo position, **(b)** PSF-width ratio (Ch1/Ch2) versus piezo position, **(c)** Axial position converted from Ch1/Ch2 photon-intensity ratio versus piezo position, and **(d)** Axial localization precision estimated from the standard deviation of the Ch1/Ch2 ratio at each z plane.

**Movie S1. Electrochemically controlled switching of Alexa 647.** Alexa 647 molecules undergo initial OFF switching under 642 nm laser (1 kW cm⁻²) illumination, followed by OFF and ON switching by applying electrochemcial potential between negative and positive values.

**Movie S2. Raw TIRF frames of Alexa 647-labeled mitochondria and CF680-labeled tubulin during EC-STORM acquisition.** Under 642 nm excitation (1 kW cm⁻²) and LASV modulation (–0.9 V to –0.2 V, 10 Hz), the frames show sparse, well-separated PSFs with clear blinking, indicating that imaging was performed predominantly in the single-molecule regime.
